# Supplementary material for: Complexome profiling of the Chlamydomonas psb28 mutant reveals TEF5 as an early PSII assembly factor
Source: Plant Cell. 2025 Mar 17;37(6):koaf055. doi: 10.1093/plcell/koaf055 (PMC12164590; doi:10.1093/plcell/koaf055)
Supplement: koaf055_Supplementary_Data [file koaf055_supplementary_data.zip › psb28_interactive.zip!/plots/intensity/qualityPlots/peptides/CAG3_Cre09.g415850.t1.2.html]

### Cre09.g415850.t1.2

Trivial name: CAG3  
SeqCoverage [median]: 64.10  
Mass: 32.69 kDa  
  
 

| Sequence | MissedCleavage | Mass [Da] | wtIntensity | muIntensity | Score |
| --- | --- | --- | --- | --- | --- |
| AALADPSNSVHQMWEYDSQTALVAR | 0 | 2759.30 | 6929.69 | 0.00 | 66.87 |
| DHVQPNLAFAPVHR | 0 | 1599.83 | 25116.76 | 21216.67 | 157.56 |
| EVVVPNK | 0 | 783.45 | 937.61 | 4079.09 | 105.27 |
| EVVVPNKHSTAFVAANANVLGNVK | 1 | 2477.34 | 1908.77 | 1984.82 | 62.68 |
| GDVNGIEVGANSNIQDNAIVHVSK | 0 | 2449.22 | 1726.62 | 0.00 | 70.92 |
| GVGAALDELGSMVQGPQGSVK | 0 | 1999.00 | 12970.64 | 21708.95 | 177.84 |
| HLEPEEASFIGK | 0 | 1355.67 | 18156.15 | 17471.47 | 200.71 |
| HSTAFVAANANVLGNVK | 0 | 1711.90 | 13321.20 | 22036.12 | 163.23 |
| IKEVVVPNK | 1 | 1024.63 | 2492.48 | 1569.01 | 122.13 |
| KPDVPVNAGQVVPAPPAAAR | 1 | 1953.08 | 34384.70 | 36850.57 | 116.51 |
| LGAGSSVWYGAVLR | 0 | 1434.76 | 8052.66 | 0.00 | 176.48 |
| SSLPAGFLFPYR | 0 | 1353.71 | 5290.69 | 0.00 | 113.71 |
| SSLPAGFLFPYRHPK | 1 | 1715.91 | 511.33 | 7153.15 | 97.72 |
|  |  |  | 131799.3 | 134069.8 |  |
